# Disulfide-Trapping Identifies a New, Effective Chemical Probe for Activating the Nuclear Receptor Human LRH-1 (NR5A2)

**DOI:** 10.1371/journal.pone.0159316

**Published:** 2016-07-28

**Authors:** Felipe de Jesus Cortez, Miyuki Suzawa, Sam Irvy, John M. Bruning, Elena Sablin, Matthew P. Jacobson, Robert J. Fletterick, Holly A. Ingraham, Pamela M. England

**Affiliations:** 1 Department of Pharmaceutical Chemistry, University of California San Francisco, San Francisco, California 94158, United States of America; 2 Department of Cellular and Molecular Pharmacology, University of California San Francisco, San Francisco, California 94158, United States of America; 3 Chemistry and Chemical Biology Graduate Program, University of California San Francisco, San Francisco, California 94158, United States of America; 4 Pharmaceutical Sciences and Pharmacogenomics Graduate Program, University of California San Francisco, San Francisco, California 94158, United States of America; 5 Department of Biochemistry and Biophysics, University of California San Francisco, San Francisco, California 94158, United States of America; University of Parma, ITALY

## Abstract

Conventional efforts relying on high-throughput physical and virtual screening of large compound libraries have failed to yield high-efficiency chemical probes for many of the 48 human nuclear receptors. Here, we investigated whether disulfide-trapping, an approach new to nuclear receptors, would provide effective lead compounds targeting human liver receptor homolog 1 (hLRH-1, NR5A2). Despite the fact that hLRH-1 contains a large ligand binding pocket and binds phospholipids with high affinity, existing synthetic hLRH-1 ligands are of limited utility due to poor solubility, low efficacy or significant off-target effects. Using disulfide-trapping, we identified a lead compound that conjugates with remarkably high-efficiency to a native cysteine residue (Cys^346^) lining the hydrophobic cavity in the ligand binding domain of hLRH-1. Guided by computational modeling and cellular assays, the lead compound was elaborated into ligands PME8 and PME9 that bind hLRH-1 reversibly (no cysteine reactivity) and increase hLRH-1 activity in cells. When compared with the existing hLRH-1 synthetic agonist RJW100, both PME8 and PME9 showed comparable induction of the LRH-1 dependent target gene *CYP24A1* in human HepG2 cells, beginning as early as 3 h after drug treatment. The induction is specific as siRNA-mediated knock-down of hLRH-1 renders both PME8 and PME9 ineffective. These data show that PME8 and PME9 are potent activators of hLRH-1 and suggest that with further development this lead series may yield useful chemical probes for manipulating LRH-1 activity in vivo.

## Introduction

Liver Receptor Homolog 1 (LRH-1, NR5A2) is among several nuclear receptors (NRs) that still lack a high affinity, selective chemical probe [1]. Early crystallographic x-ray structures showed that both the rodent and human LRH-1 ligand binding domains (LBDs) contain a large hydrophobic hourglass-shaped ligand binding cavity (800–1200 Å^3^) that can easily accommodate ligands [2–4]. Human LRH-1 LBD structures bound to either endogenous or exogenous phospholipid ligands reveal the two lipid tails buried within and occupying the entire length of the hydrophobic pocket, and the headgroup positioned at the mouth of the pocket [2, 3, 5]. In contrast, mouse LRH-1 contains a salt-bridge at the mouth of the pocket that greatly diminishes the binding of phospholipid ligands [2, 6]. Receptor-ligand interactions can greatly change the size of the ligand binding pocket, as evidenced by the contracted binding pocket observed when hLRH-1 LBD is bound to either the shorter-chain phospholipid ligand DLPC [5] or the synthetic ligand GSK8470 [7], compared to the higher-affinity phosphoinositide ligands PIP_2_ and PIP_3_ [8]. Thus for hLRH-1, standard virtual screening methods that survey a static structure might fail to capture the structural dynamics of the hydrophobic ligand binding pocket.

For several nuclear receptors, co-activator peptide recruitment to the activation function 2 (AF2) in the LBD has been successfully adapted as the primary endpoint in high throughput screening assays of compound libraries. The first reported synthetic hLRH-1 ligand, GSK8470, emerged from a high-throughput fluorescence resonance energy transfer (FRET)-based biochemical screening assay using TIF2 (NCOA2) peptide recruitment [9]. Unfortunately, GSK8470 is both unstable and insoluble making it difficult to achieve reproducible results in cellular assays [7]. Extensive modification of GSK8470 by Whitby and co-workers ultimately led to RJW100 [7], which has been used with some success in specific cellular and in vivo settings [10, 11]. In retrospect, newer biophysical data using hSF-1 strongly suggest that peptide recruitment assays might fail to discriminate between low and high affinity ligands for NR5As. Indeed, binding affinities of hSF-1 LBD for the coactivator peptide PGC-1**α** established that while significant, the absolute difference in peptide affinity in the presence of a low affinity versus high affinity phospholipid ligand (or no ligand), are quite small at, 8.5 μM and 6 μM, respectively [12]. This finding reinforces the notion that traditional screening approaches that rely on coactivator peptide recruitment assays are less effective for NR5As than perhaps for other NR subfamilies.

Here, we applied a screening strategy that identifies lead compounds based on their ability to form a disulfide bond (covalent adduct) with a naturally occurring cysteine residue that lines the ligand binding pocket of the hLRH-1 LBD. To ensure that the formation of covalent adducts is governed by the intrinsic affinity of the compounds for the ligand binding pocket, rather than their reactivity with the cysteine (thiol) sidechain, the screen is carried out in the presence of saturating concentrations of the disulfide reducing agent **β**-mercaptoethanol (BME). While this technology has been successfully employed to develop ligands for a variety of targets, it has heretofore not been applied to nuclear receptors [13, 14]. Following screening of a library of 1280 cysteine-reactive (disulfide-linked) compounds, and computational docking to visualize hit compound orientations within the hLRH-1 LBD and guide development of higher affinity ligands, elaborated non-cysteine reactive compounds were then tested in the hepatocellular carcinoma HepG2 cellular model system. Our collective results show that a lead compound identified by disulfide-trapping can be transformed into non-disulfide-linked (unreactive) biologically active ligands, illustrating the utility of the disulfide-trapping methodology for developing ligands targeting nuclear receptors.

## Results

### Disulfide-Trapping Screen

A library of 1280 disulfide-linked, low molecular weight (~300 Da) compounds (in house unpublished library) was screened against the hLRH-1 LBD. To specifically target the cysteine residue lining the ligand binding pocket (Cys^346^), the two surface-exposed cysteine residues (Cys^311^, Cys^487^) were mutated to serine. Twenty-eight compounds conjugated with high efficiency (> 2.0 standard deviations above the mean) to Cys^346^ under stringent conditions (500 μM BME) (S1 Fig). One compound (**15.31**) conjugated with remarkably high efficiency (~85%) and was selected for further development.

### Computational Modeling

**Fig 1:**
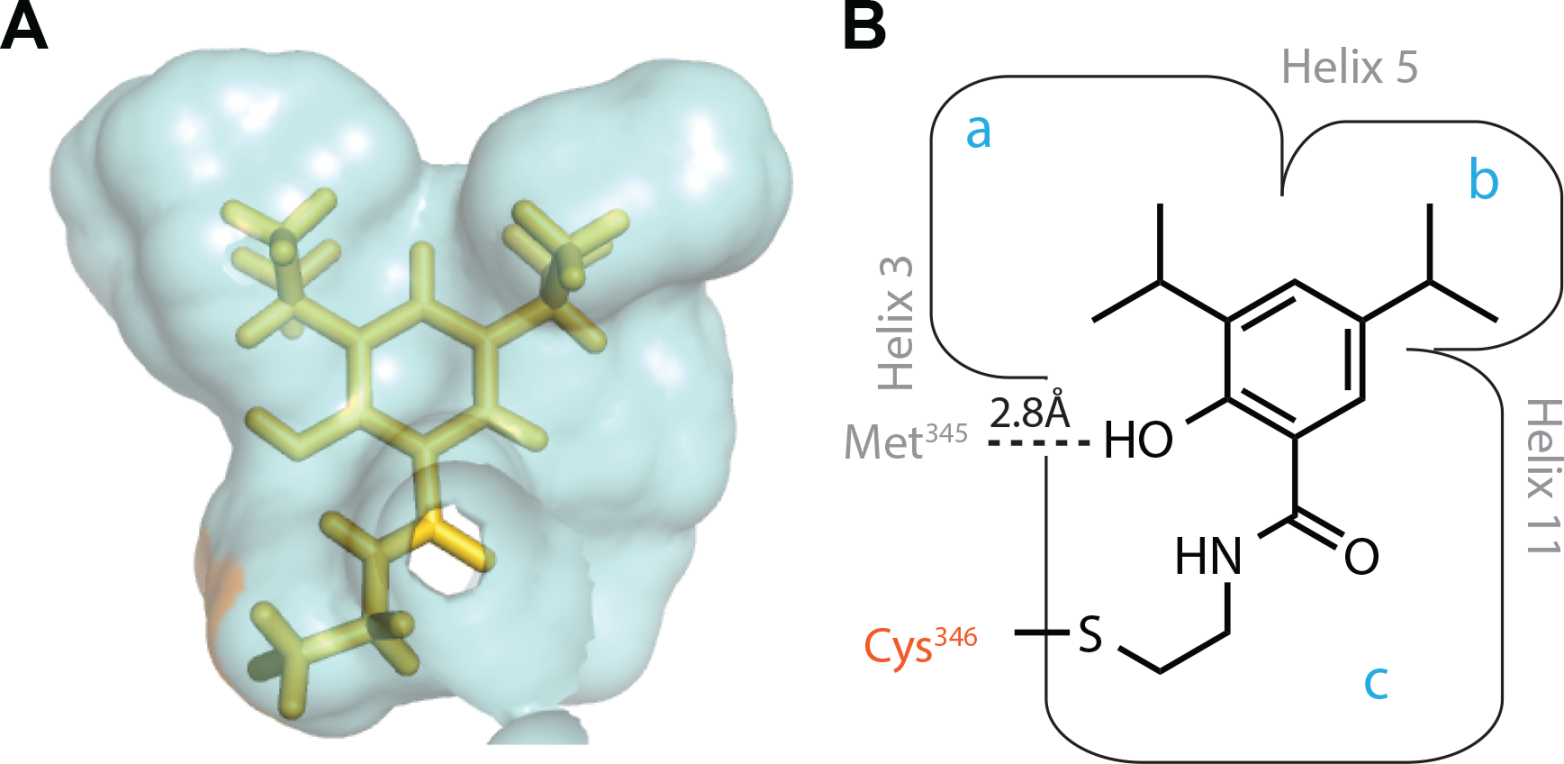
Computational model shows orientation of and space around top hit 15.31 in the hLRH-1 pocket. **A.** Computational model of top hit **15.31** covalently bound to LRH-1 through a disulfide bond to Cys^346^. Van der Walls surface (blue) represents all protein within 4.5 Å of the ligand. **B.** Structure of **15.31** and cartoon derived from the model illustrating the binding site for **15.31** is framed by helices 3, 5 and 11 and likely includes a polar interaction with the backbone carbonyl of Met^345^.

Covalent docking was used to visualize conjugated compounds bound within the hLRH-1 ligand binding domain (PDB:3PLZ).The ligand binding pocket is an hourglass shaped structure with the bottom portion nearest to the solvent. Modeling of top hit **15.31** conjugated to Cys^346^ indicated this compound occupies the top of the pocket, partially overlapping the binding site for GSK8470, and suggested a clear avenue for improving binding (Fig 1). The two isopropyl groups inadequately fill the top of the pocket, suggesting that larger substituents would improve binding. The propyl amide bends to form the disulfide link with Cys^346^; absent the disulfide the alkyl chain might be lengthened to better fill the middle of pocket. The hydroxyl group on **15.31** is located within 2.8 Å of a backbone carbonyl (Met^345^), and is the only polar contact observed in the model.

### Ligand Development

**Fig 2:**
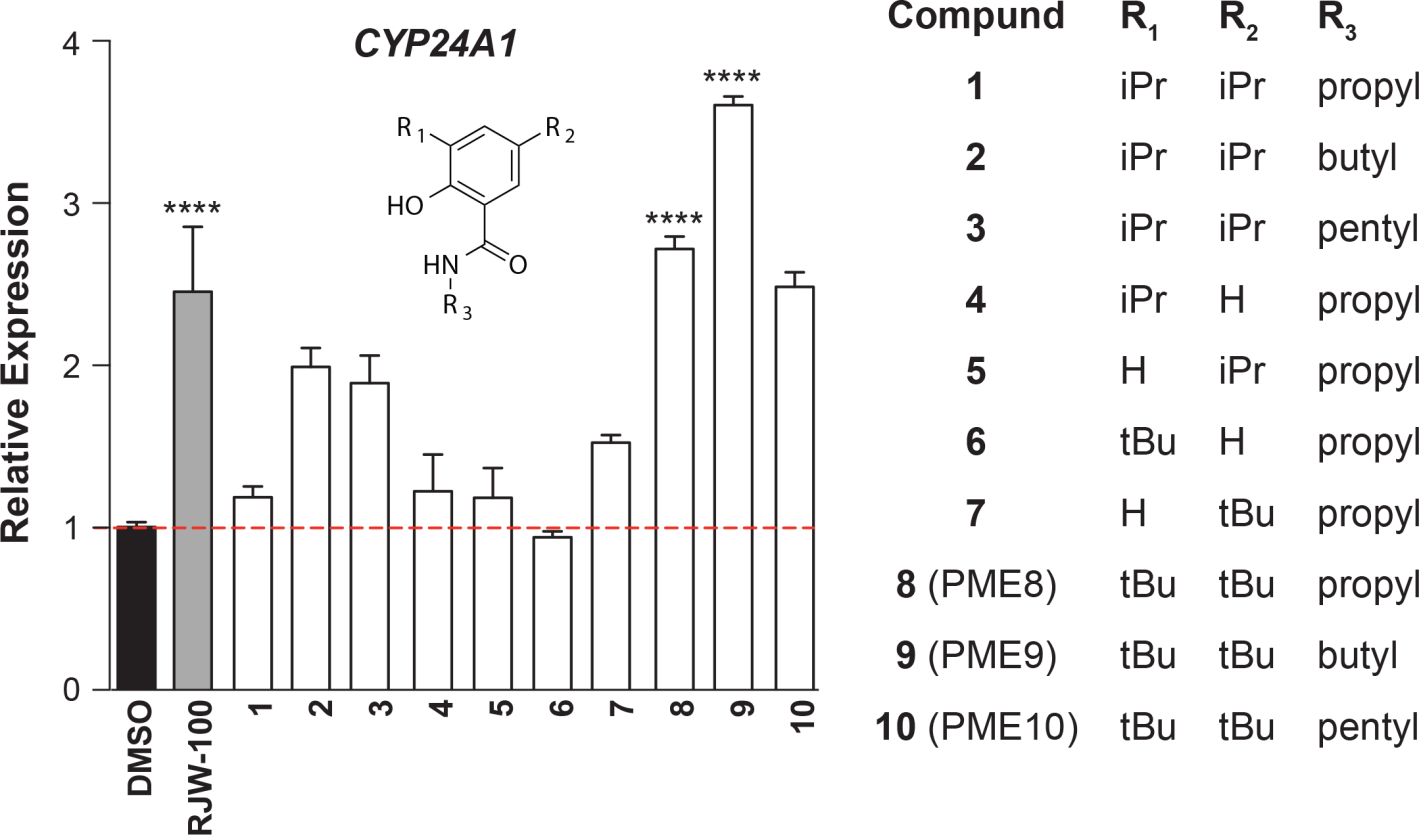
Activity of PME9 Exceeds that of RJW100 in HepG2 Cells. Relative expression of *CYP24A1* transcripts in HepG2 cells following 16 h treatment with either vehicle (DMSO) or compounds (10 μM) as listed on the X-axis. For reference, activity with the existing NR5A agonist RJW100 is shown (grey bar). For these experiments levels of hLRH-1 were low, as doxycycline (-Dox) was not added to HepG2-hLRH-1 cells (Refer to S2 Fig). Data are representative of at least three independent experiments with error bars representing SEM, *P* values = **** < 0.0001.

Based on computational models, we prepared ten non-disulfide-linked analogs of **15.31** designed to explore the ligand-receptor pharmacophore at three positions (R_1_, R_2_, R_3_ in Fig 2). Specifically, the isopropyl groups were replaced with either smaller (H) or larger (*tert*-butyl) groups and the propyl amide group was replaced with alkyl chains of longer lengths. Analogs **1**–**10** were prepared from commercially available scaffolds, by activating the carboxylic acid and subsequently coupling to alkyl amines with the indicated chain length as described in Materials and Methods.

### Cellular Assays

**Fig 3:**
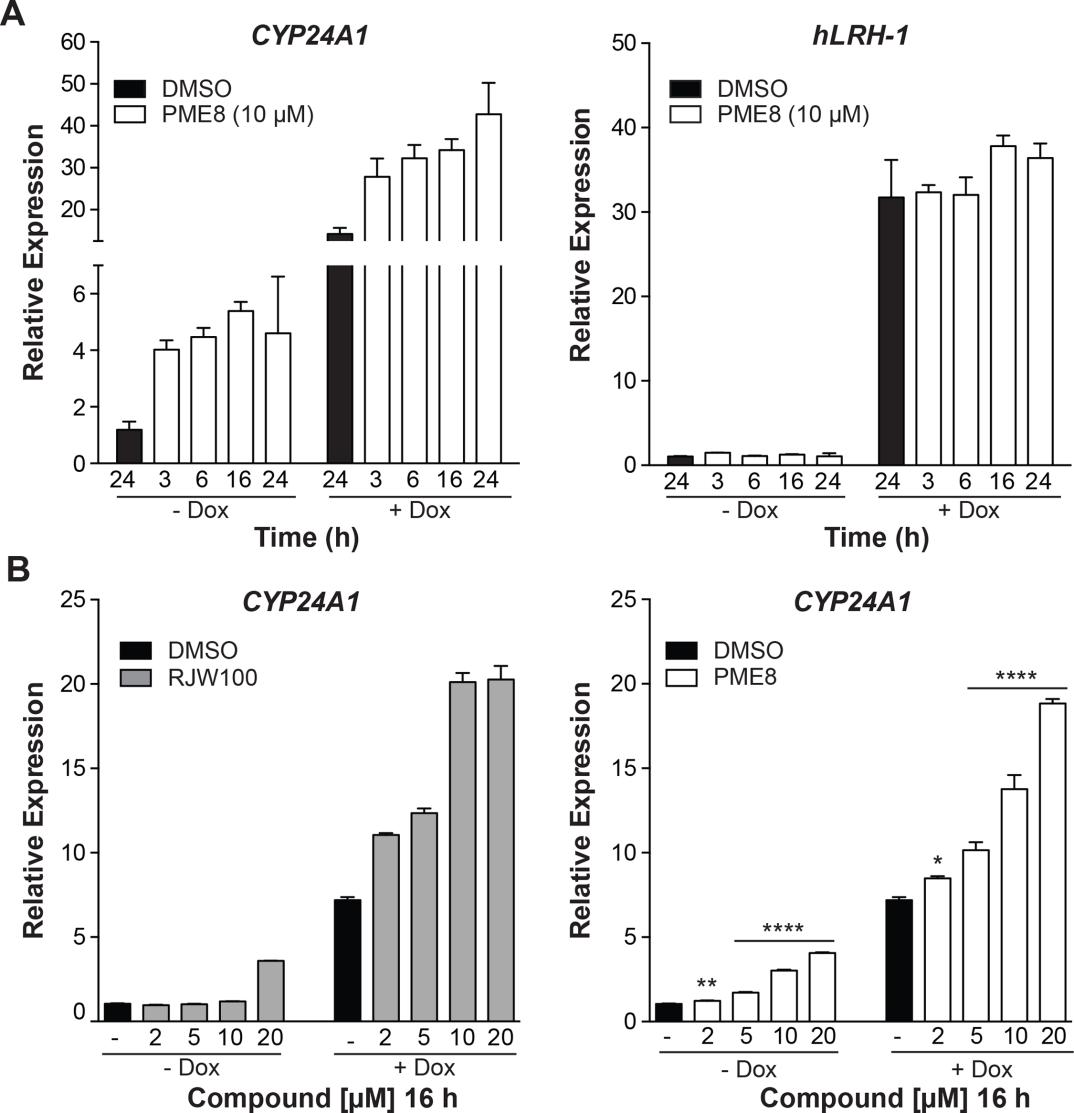
PME8 induces transcription of an hLRH-1 target gene in a both time- and dose-dependent manner with activity increasing at higher hLRH-1 levels. **A.** Relative expression levels of the hLRH-1 target gene *CYP24A1* with increasing treatment times of PME8 (10 μM) compared to the DMSO control (black bar) ranging from 3–24 h. Corresponding expression levels of *hLRH-1* transcripts in each time condition are shown in right panel without (-Dox) or with (+Dox) induction of exogenous hLRH-1. **B.** Levels of *CYP24A1* with DMSO or with increasing concentrations of RJW100 and PME8 treatment for 16 h without (-Dox) or with (+Dox) induction of exogenous hLRH-1. Data are representative of at least three independent experiments with error bars representing SEM, *P* values = **** < 0.0001.

Analogs **1–10** were tested in a hepatocellular carcinoma HepG2 cell line stably expressing hLRH-1 that can be induced to express low or high levels without or with doxycycline (-Dox or +Dox), respectively. Levels of the direct hLRH-1 target gene *CYP24A1* [15] were measured by RT-qPCR as described in Materials and Methods. A comparison of all analogs showed that compounds **8** (PME8) and **9** (PME9) (10 μM) consistently increased endogenous *CYP24A1* transcript levels 3-4-fold following a 16 h treatment time (Fig 2). The activity of PME9 exceeded that of RJW100. Importantly, when assayed in HepG2-hLRH-1 cells expressing low levels of hLRH-1, PME8 did not change hLRH-1 transcripts (Fig 3A) or protein levels (S2 Fig). Compound PME8 does increase *CYP24A1* levels in a time-dependent (Fig 3A), and dose-dependent manner with maximal activity observed at 16 h of treatment (Fig 3B).

**Fig 4:**
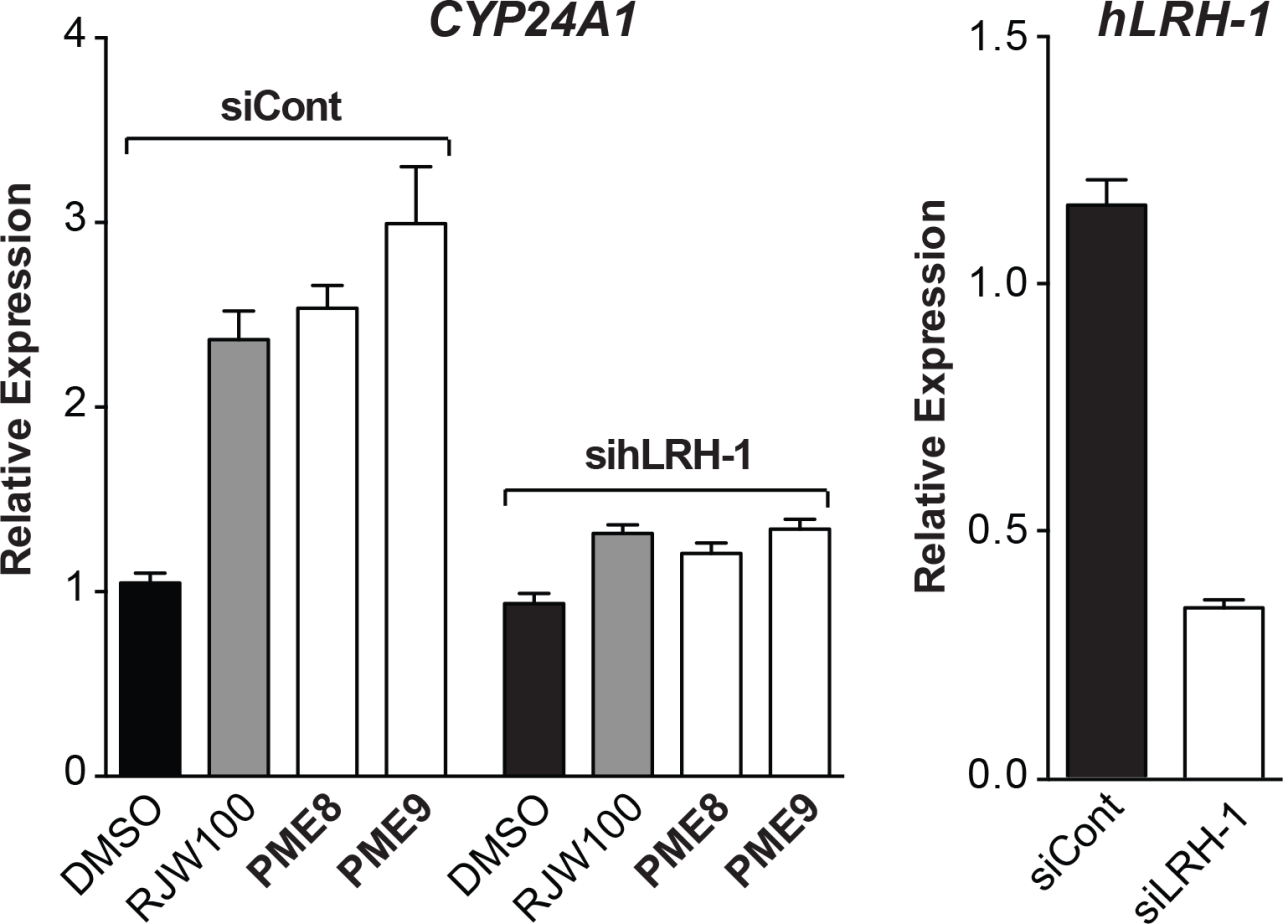
Activity of PME8 and PME9 Depends on hLRH-1. Expression of *CYP24A1* transcripts was determined in the presence of RJW100 (grey bar) or the two new compounds PME8 and PME9 (10 μM) without (siCont) or with knockdown (siLRH-1) of hLRH-1 (left panel). SiRNA-mediated knockdown of hLRH-1 in HepG2-hLRH-1 cells expressing low levels of hLRH-1 (-Dox) was carried out as described in Materials and Methods. Relative levels of *hLRH-1* transcripts after siControl or sihLRH-1 are shown (right panel).

To determine whether the activity of the two most potent compounds PME8 and PME9 are specific for hLRH-1, their activities were retested after siRNA-mediated knockdown of hRH-1 in HepG2 cells. For PME8 and PME9, as well as the control RJW100 compound, a clear dependence on hLRH-1 was observed as evidenced by the precipitous drop in *CYP24A1* expression following treatment with sihLRH-1, but not with siControl RNA (Fig 4). These ligands likely bind within the ligand binding pocket as the activity of both PME8 and PME9, as well as RJW100, decreased when tested in HepG2 cells stably expressing equivalent levels of F342W/I416W hLRH-1, a “pocket” mutant harboring two large bulky tryptophan side chains designed to fill the ligand binding cavity of hLRH-1 [2] (S3 Fig). Interestingly, although greatly reduced, there are still residual levels of *CYP24A1* after treatment with PME8 and PME9 in cells expressing this variant of hLRH-1. Consistent with these data, we observed direct binding of PME8 and PME9 to the hLRH-1 LBD using surface plasmon resonance assays (S4 Fig).

**Fig 5:**
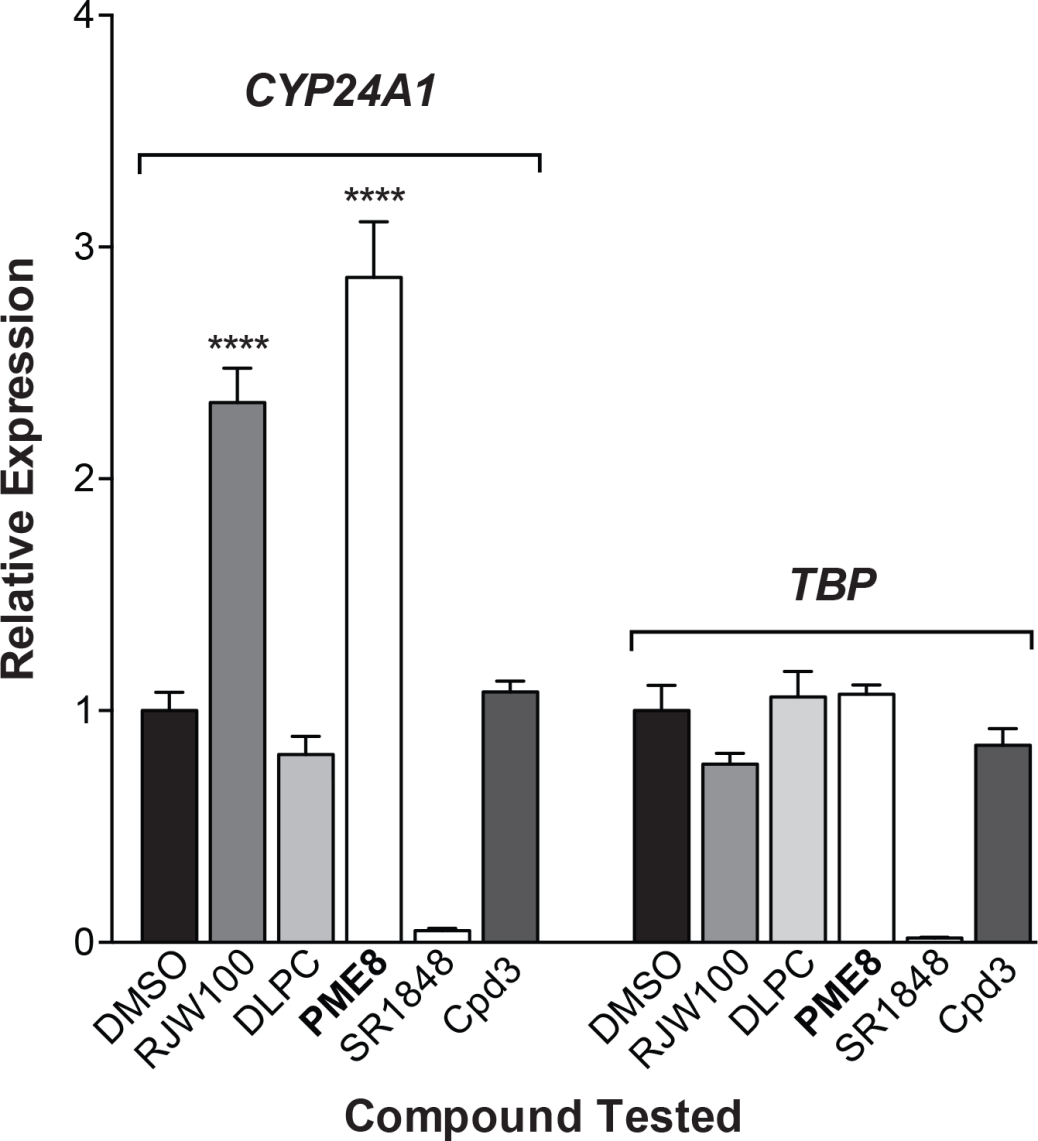
Effects of other published compounds targeting hLRH-1 compared to PME8. All published agonists and antagonists were tested in HepG2-hLRH-1 cells expressing high levels (+Dox) of hLRH-1. Activating compounds were added for 16 h relative to the DMSO control (black bars) at the following concentrations: RJW100 (10 μM) [7], DLPC (20 μM) [5], PME8 (10 μM) (bolded, this study) as well as the two putative antagonist/repressors, SR1848 (10 μM) [20] and Cpd3 (10 μM) [21]. Relative levels of the hLRH-1 target, *CYP24A1* (left set of bars) and the housekeeping gene, *TBP* (right set of bars) are shown with all values normalized to *GAPDH*. Error bars represents SEM and *P* values = **** < 0.0001.

Finally, to ask how PME8 and PME9 compare to other published compounds, we directly compared the activity of all compounds with respect to the ability to activate or repress *CYP24A1* expression in HepG2-hLRH-1 cells. For this experiment, assays were carried out under high levels of hLRH-1 (+Dox) to maximize our ability to see any repressive effects of compounds and the ligand concentrations used were based on published effective doses. Results from this experiment indicate that only one of the two published agonists, RJW100, showed the expected activity; DLPC failed to increase *CYP24A1* expression (Fig 5). Of the two published antagonist/repressors, only SR1848 decreased *CYP24A1* levels. However, SR1848 also exhibited substantial repression of the housekeeping gene, *TBP*. Similar results were also observed for another identified hLRH-1 target, *SERPINE* or *PAI1* [15, 16] (data not shown). Taken together, our findings show that PME8 and PME9 are potential new leads for creating high affinity, efficacious synthetic ligands for hLRH-1.

## Discussion

A screen of 1280 disulfide-linked compounds against hLRH-1 identified 28 hits that were trapped within the ligand binding pocket through a disulfide exchange reaction with Cys^346^. Computational modeling of these hits conjugated to Cys^346^ suggested they bind within a similar region of the pocket, at the top of the hourglass with the alkyl amide pointing toward the middle of the pocket. An advantage of covalent docking is that modeled ligand-receptor interactions (poses) are expected to be particularly accurate as the possible ligand orientations (translations and rotations) are strongly constrained by the covalent link to the protein [17, 18].

The top hit **15.31**, a substituted benzamide, was selected for further study owing to its remarkable conjugation efficiency (85%, S1 Fig) under stringent conditions (500 μM BME) and the ease with which a small, yet informative, structure activity relationship study could be carried out. The computationally modeled pose of **15.31** proved to be highly predictive (Fig 1). Increasing the size of both the R_1_ and R_2_ aryl ring substituents, from isopropyl to *tert*-butyl groups, to better fill the pocket provided compounds that were active in cells (Fig 2). The most active compound, **9** (PME9), was even more effective than RJW100, the highest affinity agonist described to date (Fig 2) [7]. The alkyl chain length (R_3_) also proved important, with *N*-butylbenzamides being more active in cells than the corresponding *N*-propyl- or *N*-pentylbenzamide compounds. This pattern, which is observed among compounds **1–3** and **8–10** (PME8-10), may reflect an inability of amide-linked alkyl groups to traverse the pocket constriction. As phospholipid tails apparently bind the length of the pocket, replacing the amide with an alkyl or ether linkage may improve the activity of compounds with longer R_3_ substituents. The hydroxyl group also contributes to efficacy, as evidenced by the difference in activity between compounds **6** and **7** (Fig 2), the latter forming a hydrogen bond with the backbone carbonyl of Met^345^ according to the computational model.

Together, these data highlight PME9 as a strong starting point for further development of hLRH-1 ligands. Specific features of the ligand binding site yet to be exploited include polar interactions with arginine and histidine residues positioned 4.8 and 3.7 Å of the R_1_ and R_2_ substituents. Moreover, the other 27 hits from the screen point to additional modifications of the benzamide scaffold expected to improve ligand efficacy, such as merging structures.

Collective data obtained from numerous in vitro and in vivo studies suggest that LRH-1 is a potential therapeutic target for several diseases including pancreatic cancer, breast cancer, colon cancer, inflammatory bowel disease and fatty liver disease. The dearth of effective synthetic probes for LRH-1 has hindered efforts to validate this nuclear receptor as a therapeutic target. Our success in developing new ligands for LRH-1 that effectively drive target gene expression is an important step and highlights the value of disulfide-trapping as a strategy for discovering lead compounds targeting nuclear receptors.

## Materials and Methods

### Disulfide-Trapping Screen

The disulfide-trapping screen was performed according to standard procedures. Purified LRH-1 (Cys311Ser, Cys487Ser) was diluted to 4 μM in a buffer containing 25 mM HEPES, 150 mM NaCl and 500 μM BME. The protein was aliquoted into a 96 well-plate (25 μL per well) and individually incubated at ambient temperature for 1 h with disulfide-linked monophores (1280 total compounds) at 150 μM. After the equilibration period, reaction mixtures were analyzed by high-throughput mass spectrometry using Waters Acquity UPLC/ESI-TQD with a 2.1 x 50mm Acquity UPLC BEH300 C4 column. The extent of labeling was determined by comparing the molecular masses of the covalently adducted protein to the molecular mass of the free protein. Hits were defined as those compounds that conjugated with efficiencies greater than two standard deviations (SD) above the mean.

### Computational Modeling

A library of 1280 disulfide-linked compounds was covalently docked to the hLRH-1 LBD. Library compounds were initially represented by their SMILES strings and then converted to 3D structures using Maestro’s LigPrep feature. In addition, LigPrep was used to identify and generate tautomers of each ligand, yielding a total of 1407 compounds for covalent docking.

The library of compounds was covalently docked to Cys^346^ in hLRH-1 using the PDB coordinate file 3PLZ, which shows the thiol sidechain oriented into the ligand binding pocket and accessible to docked ligands. PDB 3PLZ is missing the loop near Cys^346^, which connects helix 2 and helix 3. To limit potential docking artifacts, the missing H2-H3 loop residues (330–337) were manually added to the hLRH-1 sequence and the loop orientation was optimized with Prime. The predicted positions of residues within the H2-H3 loop closely aligned with other previously reported hLRH-1 crystal structures. In addition, hydrogen atoms were added and the correct charges specified using the protein preparation wizard in Maestro. Before covalently docking the library, leaving groups for the ligands and receptor were defined. Prime was used to covalently dock the 1280 disulfide-linked compounds to Cys^346^ of hLRH1. The covalent docking feature first docks each ligand to the receptor to generate a non-covalent pose. The covalent bond between the compound and the Cys^346^ side chain is then formed and the ligand pose is refined. The free energy of each ligand and the leaving groups were determined using Prime. To determine the overall binding energy the following equation was used:

$$E=E_{complex}+E_{R,LG}+E_{L,LG}-E_{receptor}-E_{ligand}$$

where *E*_*R*,*LG*_ and *E*_*L*,*LG*_ are the free energies of the receptor and ligand leaving group, respectively.

### Ligand Synthesis

All reagents were commercially available and used without further purification unless otherwise indicated. ^1^H NMR spectra were recorded using a 400 MHz Varian Inova NMR spectrometer. Chemical shifts (**δ**) are reported relative to the internal standard tetramethylsilane (TMS). Mass spectra were obtained at the University of California at Berkeley Mass Spectrometry Facility using electron spray ionization and an LTQ-FT instrument. Thin-layer chromatography was performed using silica gel 60 F_254_-coated plates (EM Science, Gibbstown, NJ). Flash column chromatography was carried out using silica gel 60 (EMD Chemical, Inc., Cincinnati, OH). All reactions were carried out under an inert atmosphere of argon unless otherwise indicated.

Compounds **1–10** were synthesized from commercially available carboxylic acids according to the following general procedures, first activating the carboxylic acid as the para-nitrobenzyl ester and then coupling to the alkyl amine to form the desired amide.

#### General procedure for preparing the activated, para-nitrobenzyl esters

To a flame-dried round bottom flask was added commercially available carboxylic acid (1 eq) as a solution in anhydrous THF (0.2 M), followed by para-nitrobenzyl chloroformate (1.1 eq) and NEt_3_ (1.2 eq) at ambient temperature. After stirring for 1 h, the mixture was poured into a separatory funnel and extracted with EtOAc against NaHCO_3_. The organic layer was dried over MgSO_4_ and then concentrated to dryness to give the crude activated ester that was used without further purification.

#### General procedure for preparing the amides

To a flame-dried round bottom flask was added the para-nitrobenzyl ester (1 eq) as a solution in anhydrous THF (0.2 M), followed by the appropriate amine (1.1 eq) and NEt_3_ (1.2 eq) at ambient temperature. After stirring overnight, the solvent was removed via rotary evaporation and **the** amide was purified to homogeneity via flash silica gel chromatography and concentrated to dryness to give the desired final product in an overall yield of 49%–65% over two steps.

#### Analytical Data for Compounds 1–10

2-hydroxy-3,5-diisopropyl-*N*-propylbenzamide (1): The title compound was synthesized in two steps from 2-hydroxy-3,5-diisopropylbenzoic acid with an overall yield of 50%; ^1^H NMR (400 MHz, CDCl_3_) δ 7.18 (s, 1H), 6.96 (s, 1H), 6.29 (s, 1H), 3.44–3.38 (m, 2H), 3.37–3.31 (m, 1H), 2.83 (hept, *J* = 7.7 Hz, 1H), 1.74–1.58 (m, 2H), 1.24–1.20 (m, 12H), 0.98 (t, *J* = 7.4 Hz, 3H); ^13^C NMR (400 Mhz, CDCl_3_) δ 170.67, 157.42, 138.20, 137.69, 128.89, 119.85, 113.22, 41.40, 33.68, 26.68, 24.22, 22.86, 22.43, 11.42. HRMS (ESI+) calcd for [M+H]+ = m/z 264.1958, found 264.1955.

***N*-butyl-2-hydroxy-3,5-diisopropylbenzamide (2):** The title compound was synthesized from 2-hydroxy-3,5-diisopropylbenzoic acid in two steps with an overall yield of 50%; ^1^H NMR (400 MHz, CDCl_3_) **δ** 7.18 (s, 1H), 6.97 (s, 1H), 6.32 (s, 1H), 3.49–3.38 (m, 2H), 3.34 (hept, *J* = 7.1 Hz, 1H), 2.82 (hept, *J* = 7.1 Hz, 1H), 1.67–1.52 (m, 2H), 1.46–1.34 (m, 2H), 1.23–1.22 (m, 12H), 0.95 (t, *J* = 7.3 Hz, 3H). ^13^C NMR (400 Mhz, CDCl_3_) δ 170.86, 157.45, 138.42, 137.85, 129.06, 119.85, 113.43, 39.68, 33.89, 31.85, 26.88, 24.43, 22.65, 20.37, 13.98. HRMS (ESI+) calcd for [M+H]+ = m/z 278.2115, found 278.2111.

**2-hydroxy-3,5-diisopropyl-*N*-pentylbenzamide (3):** The title compound was synthesized from 2-hydroxy-3,5-diisopropylbenzoic acid in two steps with an overall yield of 49%; ^1^H NMR (400 MHz, CDCl_3_) **δ** 12.50 (s, 1H), 7.19 (s, 1H), 6.97 (s, 1H), 6.32 (s, 1H), 3.45–3.39 (m, 2H), 3.35 (q, *J* = 6.9 Hz, 1H), 2.83 (p, *J* = 6.9 Hz, 1H), 1.68–1.55 (m, 2H), 1.39–1.32 (m, 4H), 1.24–1.22 (m 12 H), 0.97–0.87 (m, 3H). ^13^C NMR (400 Mhz, CDCl_3_) δ 170.61, 157.23, 138.18, 137.62, 128.83, 119.62, 113.19, 39.72, 33.66, 29.26, 29.09, 26.64, 24.19, 22.41, 22.35, 13.95. HRMS (ESI+) calcd for [M+H]+ = m/z 292.2271, found 292.2267.

**2-hydroxy-3-isopropyl-*N*-propylbenzamide (4):** The title compound was synthesized from 2-hydroxy-3-isopropylbenzoic acid in two steps with an overall yield of 51%; ^1^H NMR (400 MHz, CDCl_3_) **δ** 7.31 (d, *J* = 7.5 Hz, 1H), 7.18 (d, *J* = 7.5 Hz, 1H), 6.90–6.72 (m, 1H), 6.3 (s, 1H), 3.77–3.20 (m, 3H), 1.63 (h, *J* = 7.1 Hz, 2H), 1.22 (d, *J* = 6.9 Hz, 6H), 0.97 (t, *J* = 7.4 Hz, 3H). ^13^C NMR (400 Mhz, CDCl_3_) δ 170.79, 159.35, 138.12, 130.61, 122.70, 118.29, 113.84, 41.62, 26.66, 22.98, 22.60, 11.60. HRMS (ESI+) calcd for [M+H]+ = m/z 222.1489, found 222.1486.

**2-hydroxy-5-isopropyl-*N*-propylbenzamide (5):** The title compound was synthesized in two steps from 2-hydroxy-5-isopropylbenzoic acid with an overall yield of 50%; ^1^H NMR (400 MHz, CDCl_3_) **δ** 12.19 (s, 1H), 7.26 (d, *J* = 8.6 Hz, 1H), 7.14 (s, 1H), 6.91 (d, *J* = 8.5 Hz, 1H), 6.40 (s, 1H), 3.46–3.33 (m, 2H), 2.96–2.70 (m, 1H), 1.85–1.56 (m, 2H), 1.22 (d, *J* = 7.0 Hz, 6H), 0.99 (t, *J* = 7.4 Hz, 3H). ^13^C NMR (400 Mhz, CDCl_3_) δ 170.33, 159.80, 139.17, 132.54, 122.85, 118.69, 114.12, 41.60, 33.66, 24.34, 23.04, 11.65. HRMS (ESI+) calcd for [M+H]+ = m/z 222.1489, found 222.1486.

**3-(*tert*-butyl)-2-hydroxy-*N*-propylbenzamide (6):** The title compound was synthesized in two steps from 3-(*tert*-butyl)-2-hydroxybenzoic acid with an overall yield of 51%; ^1^H NMR (400 MHz, CDCl_3_) **δ** 7.38 (d, *J* = 7.8 Hz, 1H), 7.20 (d, *J* = 8.1 Hz, 1H), 6.75 (t, *J* = 7.8 Hz, 1H), 3.48–3.32 (m, 2H), 1.65 (m, 2H), 1.42 (s, 9H), 0.99 (t, *J* = 7.4 Hz, 3H). ^13^C NMR (400 Mhz, CDCl_3_) δ 170.78, 159.36, 138.52, 130.86, 122.40, 118.33, 113.85, 41.64, 30.25, 27.32, 22.62, 11.58. HRMS (ESI-) calcd for [M-H]+ = m/z 234.1500, found 234.1500.

**5-(*tert*-butyl)-2-hydroxy-*N*-propylbenzamide (7):** The title compound was synthesized in two steps from 5-(*tert*-butyl)-2-hydroxybenzoic acid with an overall yield of 55%; ^1^H NMR (400 MHz, CDCl_3_) **δ** 12.20 (s, 1H), 7.42 (dd, *J* = 8.7, 2.3 Hz, 1H), 7.28 (d, *J* = 2.4 Hz, 1H), 6.90 (d, *J* = 8.7 Hz, 1H), 6.47 (s, 1H), 3.49–3.34 (m, 2H), 1.72–1.58 (m, 2H), 1.28 (s, 9H), 0.97 (t, *J* = 7.4 Hz, 3H). ^13^C NMR (400 Mhz, CDCl_3_) δ 170.48, 159.41, 141.52, 131.86, 121.40, 118.34, 113.84, 41.62, 34.32, 31.61, 23.06, 11.63. HRMS (ESI+) calcd for [M+H]+ = m/z 236.1645, found 236.1642.

**3,5-di-*tert*-butyl-2-hydroxy-*N*-propylbenzamide (8):** The title compound was synthesized in two steps from 3,5-di-*tert*-butyl-2-hydroxybenzoic acid with an overall yield of 56%; ^1^H NMR (400 MHz, CDCl_3_) **δ** 7.45 (d, *J* = 1.9 Hz, 1H), 7.13 (d, *J* = 1.8 Hz, 1H), 6.34 (s, 1H), 3.49–3.33 (m, 2H), 1.65 (h, *J* = 7.6 Hz, 2H), 1.42 (s, 9H), 1.30 (s, 9H), 0.98 (t, *J* = 7.4 Hz, 3H). ^13^C NMR (400 Mhz, CDCl_3_) δ 171.46, 158.91, 139.97, 138.38, 128.87, 119.07, 113.57, 41.66, 35.41, 34.48, 31.70, 29.59, 23.08, 11.63. HRMS (ESI+) calcd for [M+H]+ = m/z 292.2271, found 292.2267.

**3,5-di-*tert*-butyl-*N*-butyl-2-hydroxybenzamide (9):** The title compound was synthesized in two steps from 3,5-di-*tert*-butyl-2-hydroxybenzoic acid with an overall yield of 50%; ^1^H NMR (400 MHz, CDCl_3_) **δ** 12.75 (s, 1H), 7.45 (s, 1H), 7.14 (s, 1H), 6.35 (s, 1H), 3.43 (m, 2H), 1.72–1.55 (m, 2H), 1.41–1.36 (m, 2H), 1.42 (s, 9H), 1.30 (s, 8H), 0.95 (t, *J* = 7.3 Hz, 3H). ^13^C NMR (400 Mhz, CDCl_3_) δ 171.19, 158.67, 139.73, 138.12, 128.61, 118.85, 113.33, 39.51, 35.17, 34.24, 31.64, 31.46, 29.35, 20.14, 13.76. HRMS (ESI+) calcd for [M+H]+ = m/z 306.2428, found 306.2423.

**3,5-di-*tert*-butyl-2-hydroxy-*N*-pentylbenzamide (10):** The title compound was synthesized from 3,5-di-*tert*-butyl-2-hydroxybenzoic acid in two steps with an overall yield of 65%; ^1^H NMR (400 MHz, CDCl_3_) **δ** 12.76 (s, 1H), 7.45 (s, 1H), 7.14 (s, 1H), 6.36 (s, 1H), 3.90–2.71 (m, 2H), 1.77–1.50 (m, 32), 1.42 (s, 9H), 1.39–1.31 (m, 4H), 1.30 (s, 9H), 0.91 (t, *J* = 6.5 Hz, 3H). ^13^C NMR (400 Mhz, CDCl_3_) δ 171.18, 158.67, 139.73, 138.12, 128.61, 118.87, 113.34, 39.79, 35.17, 34.24, 31.46, 29.36, 29.27, 29.10, 22.36, 13.96. HRMS (ESI+) calcd for [M+H]+ = m/z 320.2584, found 320.2580.

#### Cell lines and Quantitative Real-time Polymerase Chain Reaction (RT-qPCR) Assay

HepG2 3G stable cells expressing 3X-Flag-tagged WT and pocket mutant (F342W/I416W) hLRH-1 were generated as described [15] after insertion into the doxycycline (Dox)-inducible pTRE 3G vector (Clontech) to generate HepG2-hLRH-1 stable cell lines. The parental TET-On 3G HepG2 cell line was a generous gift from Dr. Stephen Hand. Induction of low or high levels of hLRH-1 was achieved by addition of DMSO or 500 ng/ml Dox, respectively.

For RT-qPCR assay, cells (5 x 10^5^) were plated on 24-well plates in 0.5 mL of media. The following day, fresh media was applied with compound or DMSO control. After an overnight treatment or indicated period of time, cells were washed with ice-cold 1 X PBS and total RNA was isolated by Trizol (Life Technologies). DNase-treated total RNA was used to generate cDNA using High-Capacity cDNA Reverse Transcription kits (Life Technologies). RT-qPCR was performed with SYBR (Biotool) and data analyzed essentially as described [19]. Sequences for all primer pairs used for qPCR reactions are listed in S1 Table. For siLRH-1 knockdowns, siLRH-1 (GS2494) and non-silencing control (SI03650318) siRNA were purchased from Qiagen. SiRNAs were used at a final concentration of 5 nM and reverse-transfected into HepG2-hLRH-1 stable cells by RNAiMax (Life Technologies) for 72 h. Knock-down was confirmed after assaying hLRH-1 transcripts by RT-qPCR.

#### Surface Plasmon Resonance Assays

Data were collected on a BiaCore T100 instrument. The biotin CAPture kit (GE Healthcare Life Science) was used as directed for chip preparation and immobilization of protein. In brief, the CAP chip was first conditioned with 3 x 1 min injections of the provided regeneration solution (6 M Guanidine HCL, 0.25 M NaOH). CAP reagent was then captured on flow cells 1 and 2 to a final density of ~ 2500 response units using capture buffer (25 mM HEPES, pH 7.4, 150 mM NaCl). Avi-tagged LRH-1 was then directly injected exclusively on to flow cell 2 achieving a final density of ~ 1000 response units (capture buffer). Flow cell 1 served as the reference flow cell and flow cell 2 served as the active flow cell. A titration series for each fragment was generated as a 0.6x dilution series starting from 300 μM. All samples were made to match the running buffer used in the experiment as closely as possible (capture buffer + 3% DMSO, + 0.05% Tween-20, 250 μM TCEP). Data was collected at 25°C with a flow rate of 30 μL/min. Data processing included referencing to both the reference flow cell as well as a buffer injection. Equilibrium binding constants were determined by nonlinear regression analysis utilizing a simple 1:1 binding model. Steady state values used to generate dose-responses curve correspond to the response units achieved 20 seconds after injection.

## Supporting Information

S1 Fig Disulfide-trapping screen and structure of top hit 15.31. (**A**) Cartoon depicting the screening strategy. Each molecule in the library consists of two elements: a monophore (circle, square, oval), which is the unique chemical entity, and a common linker region (-S-S-R) that can undergo disulfide exchange with thiols, including cysteine side chains. Library compounds are individually incubated with the target protein (hLRH-1 LBD) under high reductant conditions (500 μM BME), such that disulfide exchange is rapid. Compounds with high intrinsic affinity for a binding site near a cysteine residue (Cys^346^ in hLRH-1) have superior residence time and thus encourage the lasting formation of a disulfide bond between the protein thiol and compound thiol. Compounds with weak inherent affinity are readily reduced off. (**B**) Graph showing the conjugation efficiency for each of 1280 compounds in the library to hLRH-1. Notably, the disulfide-trapping screen was carried out against hLRH-1 bound to a phospholipid (phosphatidylethanolamine) co-expressed with the receptor in bacteria. As was observed by Whitby and coworkers (Whitby et al., 2006), small non-polar molecules are clearly able to displace bound bacterial phospholipid ligands from the pocket.
(EPS)

S2 Fig Expression of hLRH-1 is unaffected by PME8. Expression of hLRH-1 detected in Western blots using anti-Flag antibody is shown in HepG2 cells expressing low (-Dox) or higher levels (+Dox) of hLRH-1. All three sumoylated species of hLRH-1 (SU-LRH-1) are observed after Induction of high levels of hLRH-1, as indicated by arrows.
(EPS)

S3 Fig Activity of RJW100, PME8 and PME9 depends on an intact hLRH-1 ligand binding pocket. Expression of *CYP24A1* transcripts in the presence of DMSO (black bar), RJW100 (grey bars), PME8 and PME9 (white bars) at 10 μM for 16 h treatment in HepG2-hLRH-1 cells expressing low levels of wild type hLRH-1 or the hLRH-1 pocket mutant F342W/I416W variant as described in Materials and Methods.
(EPS)

S4 Fig PME8 and PME9 bind directly to the hLRH-1 LBD. (Left) Raw surface plasmon resonance sensorgrams (time vs response) for PME8 and PME9 show direct binding to biotinylated apo hLRH-1 LBD. (Right) Dose-response curves (log ligand concentration vs response) for PME8 and PME9 with apparent dissociation constants determined using steady state values at 20 s post injection (red dots on sensorgrams) and a 1:1 binding model.
(EPS)

S1 Table RT-qPCR Primer Sequences. (EPS)
